# Functional microneedles for wearable electronics

**DOI:** 10.1002/SMMD.20220023

**Published:** 2023-02-12

**Authors:** Xiaoxuan Zhang, Minhui Lu, Xinyue Cao, Yuanjin Zhao

**Affiliations:** ^1^ Department of Rheumatology and Immunology Nanjing Drum Tower Hospital School of Biological Science and Medical Engineering Southeast University Nanjing China; ^2^ Oujiang Laboratory (Zhejiang Lab for Regenerative Medicine, Vision and Brain Health) Wenzhou Institute University of Chinese Academy of Sciences Wenzhou Zhejiang China

**Keywords:** bio‐detection, microneedle, sensing, self‐powering, wearable electronics

## Abstract

With an ideal comfort level, sensitivity, reliability, and user‐friendliness, wearable sensors are making great contributions to daily health care, nursing care, early disease discovery, and body monitoring. Some wearable sensors are imparted with hierarchical and uneven microstructures, such as microneedle structures, which not only facilitate the access to multiple bio‐analysts in the human body but also improve the abilities to detect feeble body signals. In this paper, we present the promising applications and latest progress of functional microneedles in wearable sensors. We begin by discussing the roles of microneedles as sensing units, including how the signals are captured, converted, and transmitted. We also introduce the microneedle‐like structures as power units, which depend on triboelectric or piezoelectric effects, etc. Finally, we summarize the cutting‐edge applications of microneedle‐based wearable sensors in biophysical signal monitoring and biochemical analyte detection, and provide critical thinking on their future perspectives.

## Graphical abstract

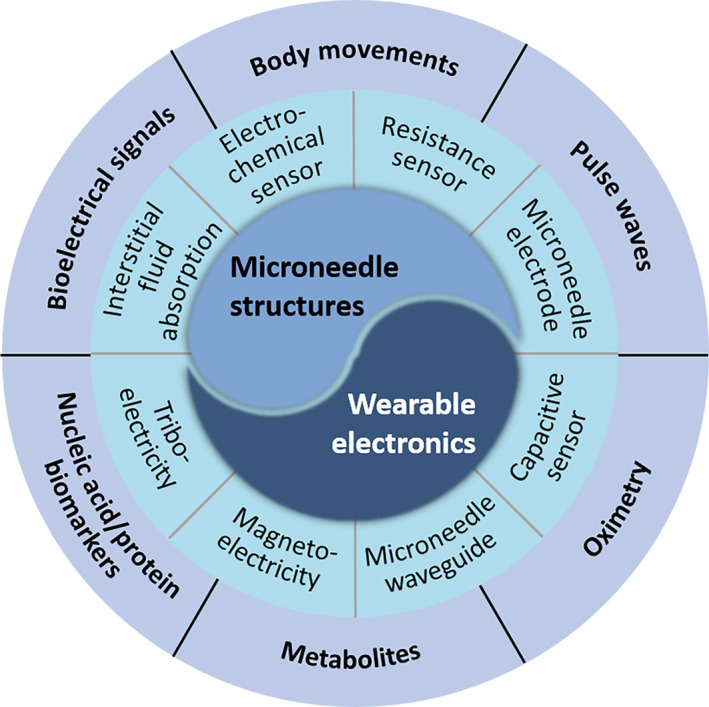

The integration of microneedle structures can significantly improve the performances of wearable electronics and greatly promote their developments. This review outlines different types of functional microneedle‐integrated wearable electronics, including electrochemical biosensors, capacitive biosensors, triboelectric biosensors and so on. The most advanced applications of these novel wearable electronics, such as gesture monitoring and biomarker detecting, are also discussed.

1

**Key points**

- Microneedle structures can act as sensing units to contribute to interstitial fluid extraction, electrochemical detection, and the construction of electronic biosensors.
- The shape geometry and flexibility of microneedle structures enable them to serve as power units for magnetoelectric or triboelectric sensors.
- Microneedle‐integrated wearable electronics are playing an important role in monitoring biophysical signals as well as detecting biochemical analytes in tissue interstitial fluids.

## 2 INTRODUCTION

Wearable electronics generally refers to integrated and smart analytical devices worn by users that have direct/indirect contact with the human body, collect human health information, and provide in‐time readable feedback to users.^1^, ^2^, ^3^, ^4^, ^5^ Basically, a wearable sensor contains three major building blocks (a sensing unit for interfacing, sampling, signal recognizing and information amplifying, a decision‐making unit for data collecting, analyzing and processing, and a power unit for supplying power) and other auxiliary parts for assembly, sealing, and supporting.^6^, ^7^, ^8^ Benefitting from their convenient usage, small sizes, high integration, and good practicability, wearable sensors are now receiving much attention from both scientific research studies and commercial markets.^9^, ^10^, ^11^, ^12^, ^13^, ^14^ During their initial phase, wearable sensors focus on monitoring biophysical signals such as locomotion, heart rate, body temperature, and so on.^15^, ^16^ As they continuously develop, wearable sensors that can detect biochemical analytes in a non‐ or minimally invasive manner have emerged.^17^, ^18^, ^19^, ^20^, ^21^, ^22^ These wearable sensors also come in diverse forms and structures, including patches,^19^, ^23^ films,^24^ tattoos, and textiles,^25^, ^26^ together with microneedles,^23^, ^27^, ^28^ wrinkles, and micro‐protrusions.^6^, ^29^

Recently, an increasing number of efforts have been devoted to generating wearable sensors with microneedle structures. Microneedles are generally defined as an array of microscale cone/pyramid protuberances that can overcome skin barriers and contact interstitial fluids without touching blood capillaries and nerve endings.^30^, ^31^, ^32^ The integration of microneedle structures can benefit wearable sensors in many aspects. On one hand, due to their skin penetration capacity, minimal invasiveness, and harmlessness,^33^, ^34^ these microneedles greatly improve the sampling accuracy and efficiency of wearable sensors. By further modifying the microneedles with aptamers or complementary substances, microneedle‐based wearable sensors can realize specific biomarker capture, recognition, and detection in situ.^23^, ^28^, ^35^ On the other hand, the high specific surface area of microneedle structures^36^, ^37^, ^38^ is helpful for wearable sensors to sense and identify slight signals, thus greatly improving their sensitivity. Specially, when serving as a part of the power unit, the microneedle structures are conducive to charge enrichment and power supply.^39^

**FIGURE 1:**
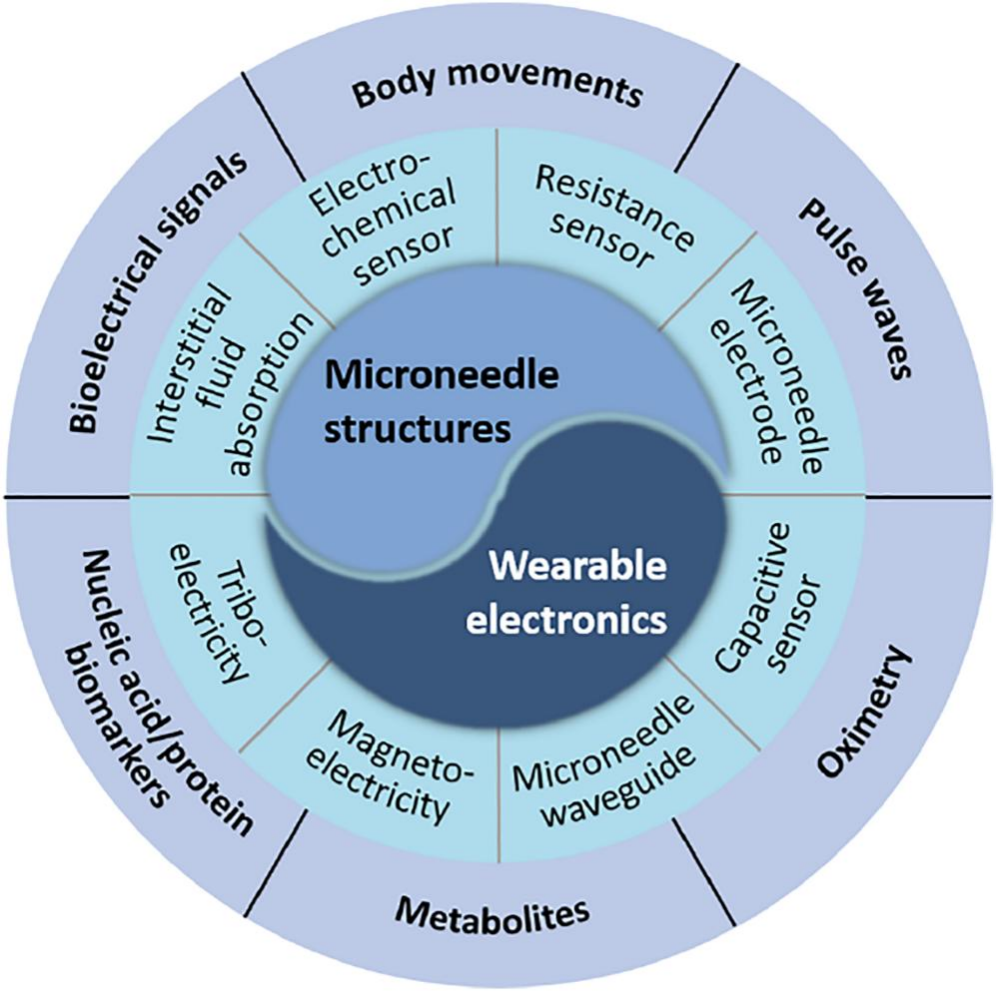
Schematic illustration of different types of microneedle‐integrated wearable electronics and their applications in healthcare and disease diagnosis.

In this paper, we provide a concise summary of the advantages and up‐to‐date applications of functional microneedle‐integrated wearable electronics (Figure 1). We start by discussing the roles of microneedle structures as different units. In Section 2, we investigate the mechanism of microneedle‐based sensing units, where the microneedle structures can facilitate signal capture, conversion, and transmission based on absorption, target‐aptamer recognition, piezoresistive effects, iontophoretic effects, and so on. Besides, in Section 3, the microneedle structures have been incorporated into triboelectric sensors, magnetoelectric sensors, etc. to act for the power units. In Section 4, we talk about the most recent progress of applying microneedle‐based wearable sensors to biophysical signal monitoring and biochemical analyte detecting. We also list the most common physical or chemical indicators that can be detected by these sensors. In the last section, we provide critical envisions on the current challenges, improvement directions, and future perspectives of microneedle‐integrated wearable sensors.

## 3 MECHANISM OF MICRONEEDLE‐BASED SENSING UNITS

Microneedle structures own many properties that make them competent as sensing units. To begin with, benefitting from their high‐narrow shapes and fine‐tuned dimensions, microneedles can overcome skin barriers in a minimally invasive, harmless, and painless manner^40^, ^41^, ^42^ and thus can fully contact tissue interstitial fluids and accumulate the biomarkers. With further integration with specific aptamers and serving as electrochemical electrodes,^22^, ^43^ the microneedles can smartly capture the desired analytes, timely reflect the related information, and realize specific and accurate sensing and detection in situ. In addition, microneedle structures are easy to fabricate, versatile in material compositions, and compatible with different kinds of decorations.^44^, ^45^, ^46^, ^47^ These, together with their high specific surface area, help to enhance the signal acquisition and sensing functions of electrical sensors,^48^, ^49^ such as resistance‐based, conductance‐based, capacitance‐based sensors, etc.

### 3.1 Interstitial fluid absorption

With proper material compositions and special microstructures, microneedles can efficiently absorb tissue interstitial fluids and provide information for the downstream electronic module. For example, Gao et al. used swellable silk fibroin hydrogel as the microneedle materials and designed them into an inverse opal photonic crystal microstructure.^50^ This microneedle device was also integrated with a microfluidic channel module and a micro electro circuit module. When the device was applied, the tissue interstitial fluids would pass through the porous microneedles and diffuse along the microfluidic channels for analysis.

**FIGURE 2:**
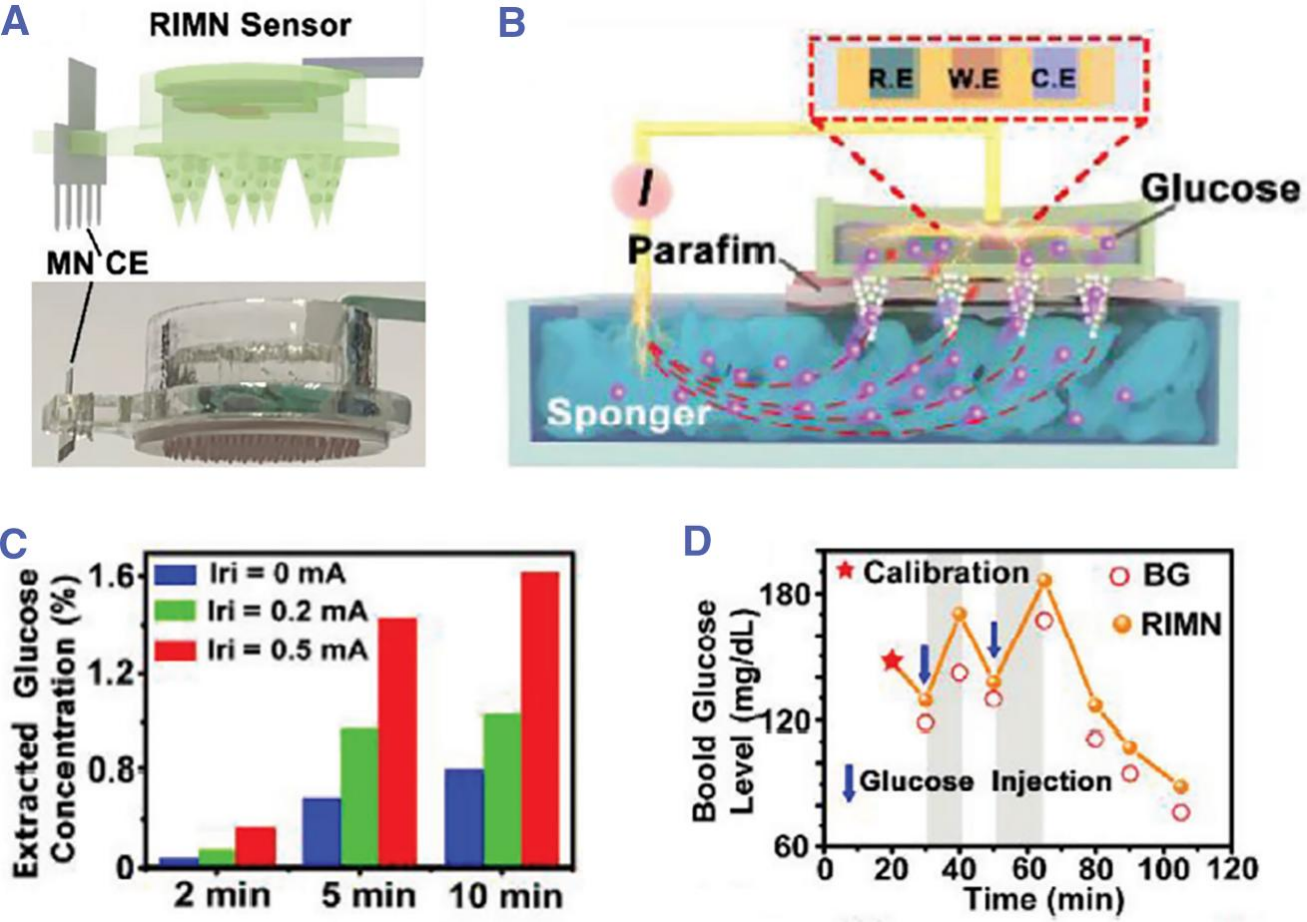
Reverse iontophoresis‐based microneedle sensors for interstitial fluid glucose extraction and detection. (A) Schematic and optical image of the microneedle sensor. (B) Schematic of the glucose extraction and detection process. (C) Extraction efficiency of glucose under different iontophoretic currents (0, 0.2 and 0.5 mA) and time (2, 5 and 10 min). (D) Rat glucose concentrations recorded by the microneedle sensor in comparison with blood glucose levels measured by standard methods. Reproduced under terms of the CC‐BY license.^52^ Copyright 2021, The Authors, published by John Wiley and Sons.

Notably, to further improve the extraction efficiency of biomarkers in interstitial fluids, some ancillary techniques such as iontophoresis are involved. By inducing charged flow and electro‐osmotic flow, iontophoresis can not only electrically release substances across the skin but also draw molecules from interstitial fluids to the skin surface.^51^ The latter is termed as reverse iontophoresis. Based on this principle, Xie and his coworkers invented a wearable microneedle‐based closed‐loop electronic system that could both monitor glucose levels and responsively deliver insulin for blood glucose control and diabetes treatment.^52^ For the glucose sensing part, reverse iontophoresis extracted a large quantity of interstitial fluid glucose to the sensor chamber, where a three‐electrode system would electrochemically detect the glucose content (Figure 2A,B). By detecting glucose in artificial skin tissues, it was demonstrated that the glucose extraction efficiency of this sensor increased with increasing iontophoretic currents and iontophoresis durations (Figure 2C). Besides, the dynamic glucose signal of healthy rats recorded by this sensor was consistent with that measured by standard glucose test strips, indicating the accuracy and practicability of this microneedle‐based sensor (Figure 2D).^52^

### 3.2 Electrochemical integration

Although the majority of current wearable electrochemical electronics focuses on perspiration sensing,^53^, ^54^ increasing attention has been drawn to tissue interstitial fluids as sensing sources in recent years. This can be attributed to the similarity in composition and concentration between interstitial fluids and blood.^55^ Microneedles are widely used as electrochemical electrodes in these interstitial fluid sensors with solid microneedles coming first, which are easy and cheap to fabricate and process. By coating stainless steels or silicon with conductive materials, such as platinum,^56^ gold,^57^ conductive polymers,^58^ etc., different microneedle electrodes can be fabricated to monitor molecules in skin interstitial fluids, including cations,^59^ hydrogen peroxide,^60^ glucose,^61^, ^62^ nucleic acids,^63^ proteins,^64^ and so on.

**FIGURE 3:**
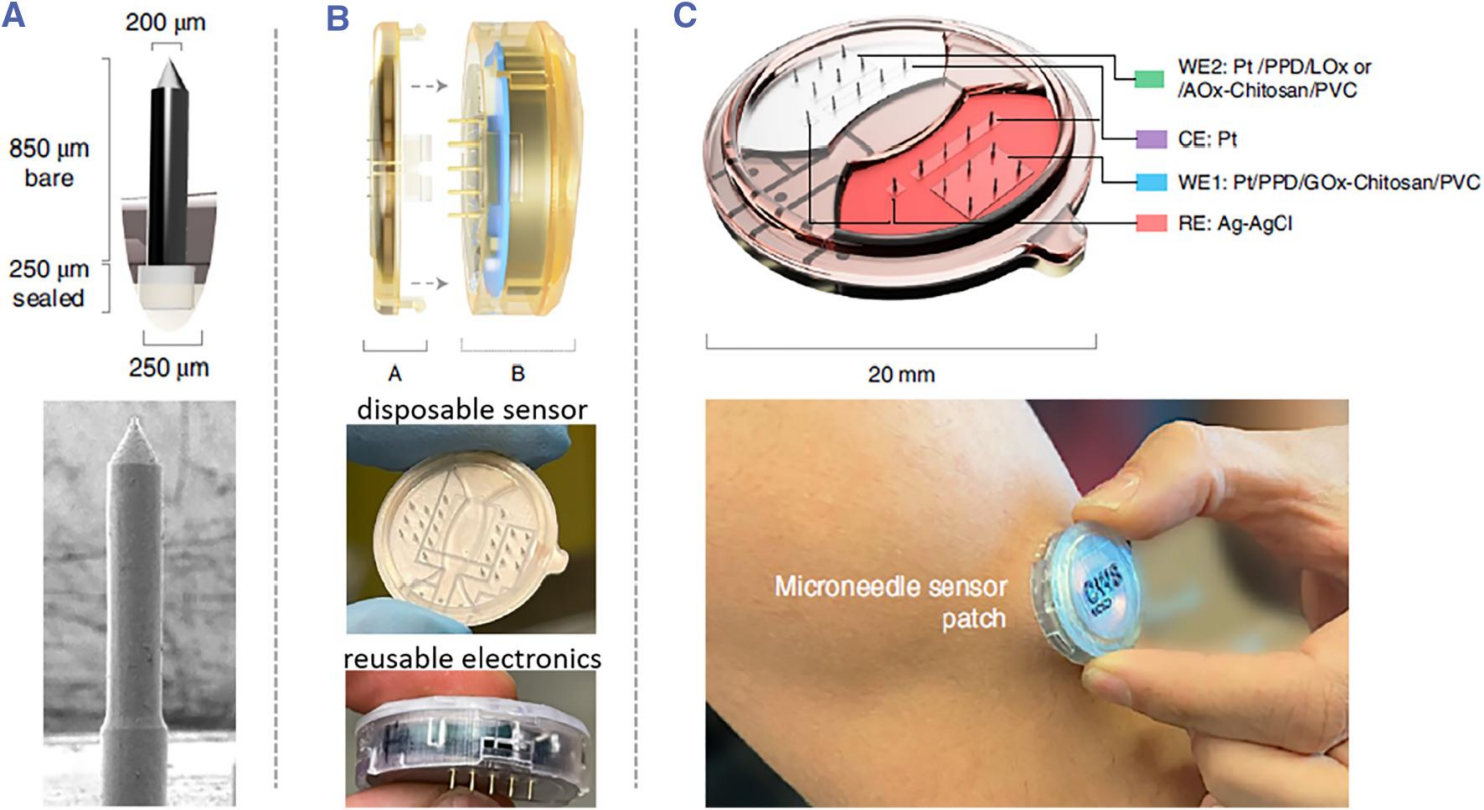
Wireless microneedle‐integrated sensing system for electrochemically detecting metabolites of human subjects. (A) Schematic and scanning electron microscope (SEM) images of the microneedles. (B) Schematic and optical images of two major parts of the sensing system: a disposable sensor part (A) and a reusable electronics part (B). (C) Schematic and optical images of the integrated sensing system. Reproduced with permission.^65^ Copyright 2022, The Authors, published by Springer Nature.

For example, to detect small‐molecular physiological indexes in interstitial fluids, Tehrani et al. developed a wireless, fully‐integrated sensing system by employing poly(methyl methacrylate) with good biocompatibility and mechanical strength as the microneedle material (Figure 3).^65^ Poly‐o‐phenylenediamine was then deposited on these microneedle electrodes to reject interference, which was successively coated by chitosan polyelectrolyte mingled with different oxidase enzymes as well as hydrophobic polyvinyl chloride for limiting glucose diffusion and antifouling. It was found that such a sensing system could conduct both single‐analyte and dual‐analyte measurements, and report real‐time, continuous and reliable data of metabolites (lactate, glucose, or alcohol) of human volunteers during daily activities.^65^

The microneedle‐integrated electrochemical sensor can also detect disease‐related biomarkers for timely diagnosis. For instance, by combining conductive microneedles with CRISPR‐Cas9‐activated graphene biointerfaces and reverse iontophoresis, sepsis, herpes, and kidney transplantation cell‐free DNA (cfDNA) in interstitial fluids could be sensitively monitored for a long term.^63^ In another example, immobilized catechol, the substrate of the tyrosinase cancer biomarker, was filled in a hollow microneedle to construct electrochemical sensors.^64^ These sensors behaved well in detecting tyrosinase and thus were significant for melanoma screening. Additionally, drugs such as tobramycin sulfate were proved to be electrochemically detected by nucleic acid aptamer‐decorated gold microneedles.^66^ Based on the high affinity between aptamers and targets, drug concentrations in interstitial fluids could be continuously monitored.

### 3.3 Resistance‐based sensor

**FIGURE 4:**
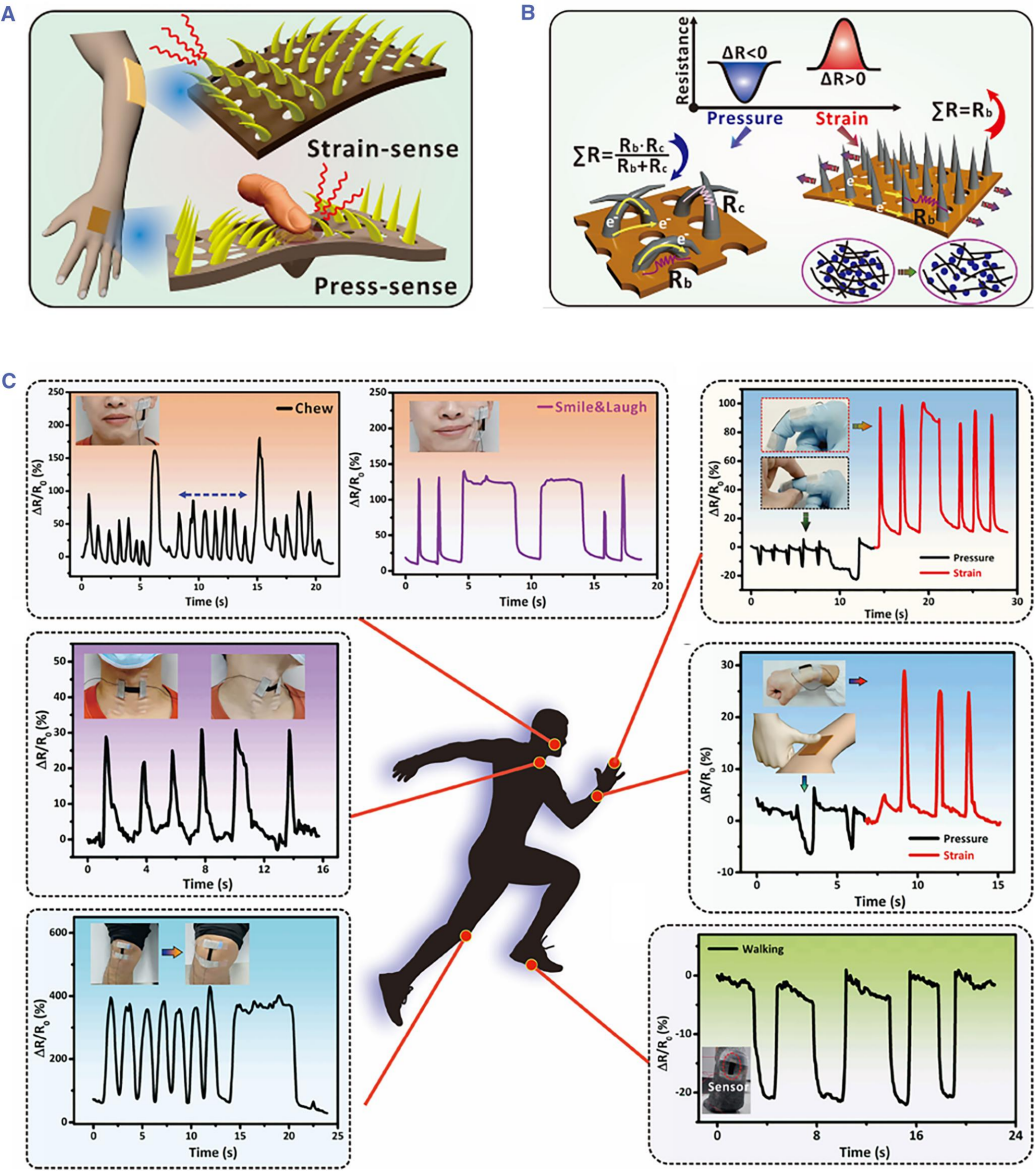
Microneedle‐integrated electronic skins with varying resistances under different forces. (A) Schematic of the electronic skin under strains or presses. (B) Schematic of the mechanism for force‐induced resistance changes. (C) Results of body movement detection via the electronic skin. Reproduced with permission.^71^ Copyright 2022, American Chemical Society.

A microneedle‐assisted, resistance‐based sensor typically contains a microneedle‐like conductive layer that is in contact with the electrode substrate.^67^ These microneedle‐like layers can magnify slight pressure signals by generating resistance changes and are thus good at sensing in low‐pressure regimes. Elastomer (e.g. polydimethylsiloxane) covered by conductive materials (e.g. poly(3,4‐ethylenedioxythiophene): poly(styrenesulfonate),^67^ gold nanowires,^68^ MXene nanoflakes^69^ and carbon black^70^) or mingled with conductive substances (e.g. carbon nanotube,^71^ carbon black^72^ and nano‐copper^72^) is usually used to constitute the microneedle‐like layers. Up to now, researchers have applied the resistance‐based sensors to monitor wrist pulse,^68^ recognize voice,^69^ detect body movements^72^ and so on. For example, a microneedle‐integrated electronic skin was presented for sensing both pressure and strain forces (Figure 4A).^71^ When pressure was applied, the contact points between microneedles and the substrate would increase, resulting in more parallel conductive circuits and decreased resistance; while an applied strain would bring about fewer contact points and conductive paths, increasing the resistance (Figure 4B). Depending on this mechanism, the proposed electronic skin could sensitively recognize facial expressions (chewing, smiling, and laughing) and monitor whole‐body movements (finger, wrist, neck, foot, and knee), as shown in Figure 4C.^71^

### 3.4 Conductance‐based sensor

**FIGURE 5:**
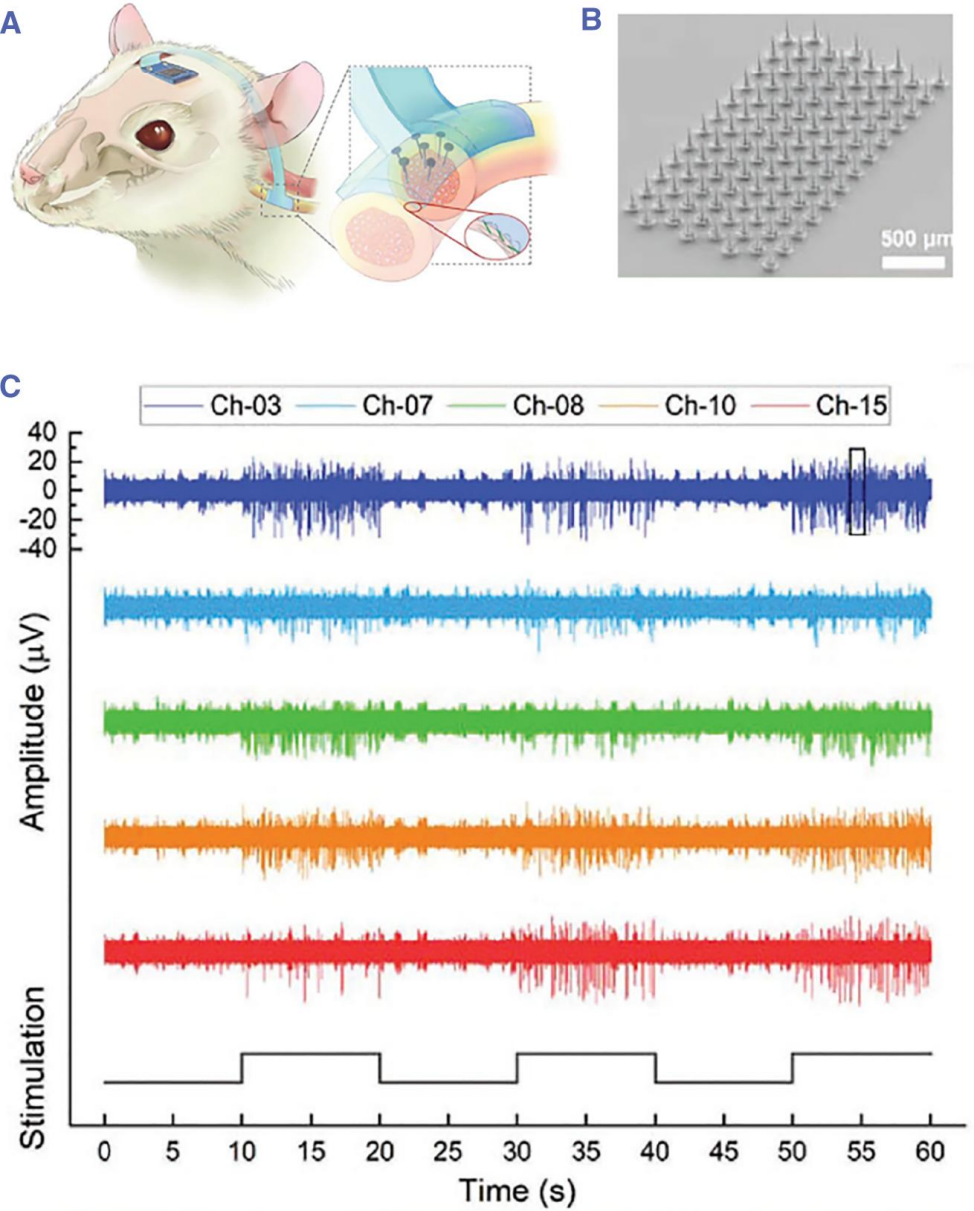
Microneedle electrodes for mapping peripheral nerve signals. (A) Schematic of applying the device to the rodent autonomic nerve. (B) SEM image of the microneedle electrodes. (C) Rodent peroneal nerve signals recorded by five different microneedle electrodes. Reproduced with permission.^73^ Copyright 2022, John Wiley and Sons.

In general, microneedles serve as tissue‐penetrating electrodes for a conductance‐based sensor.^73^ They are required to have high conductivity and the ability to reach deep tissues to precisely collect electrophysiological signals. The materials for these microneedle electrodes can be stainless steel,^74^ silicon,^75^ and conductive metals (chromium and gold).^76^ Besides, various signals, such as electromyography,^74^ electroencephalography,^76^ and even broadband brain activity mapping,^75^ have been successfully recorded by the microneedle electrodes. For example, Seymour and coworkers fabricated a peripheral nerve mapping device coupled with a 140‐μm tall silicon microneedle electrode array (Figure 5A,B).^73^ This system was constructed via a series of microelectromechanical system technologies, including deep reactive ion etching, sputtering, and thermal oxidation. In the attempt to obtain intrafascicular recording from the somatic peroneal nerve, the microneedle‐coupled device was implanted into the rodent peroneal nerve, and cutaneous brushing stimulation was applied to the hind leg. The signals from five different channels showed that the peak‐to‐peak amplitude varied from 23 to 40 μV and that the average signal‐to‐noise ratio was about 9.74 dB (Figure 5C).^73^

### 3.5 Capacitance‐based sensor

**FIGURE 6:**
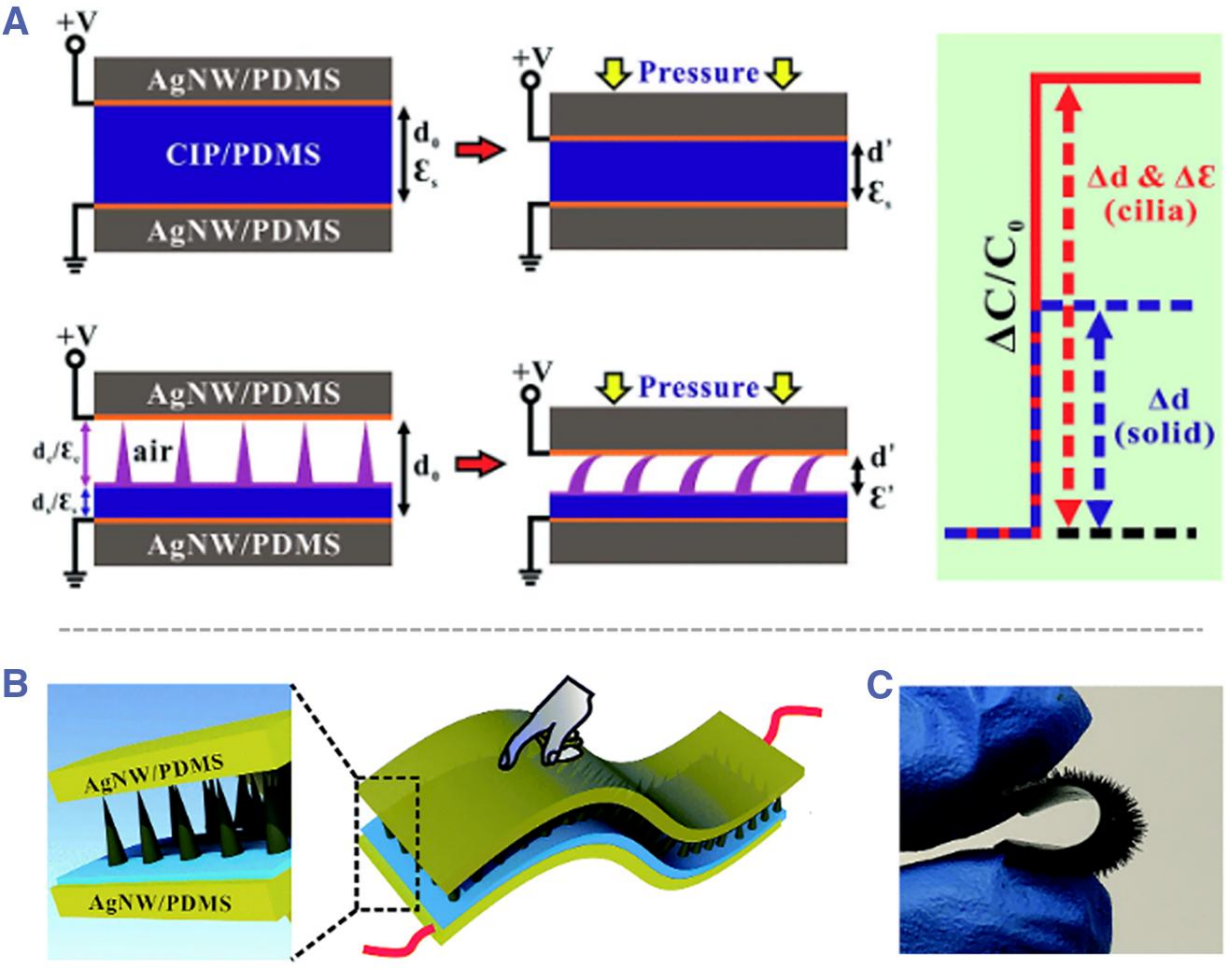
Microneedle as the dielectric layer for improving capacitance‐based sensor performances. (A) Schematics of the enhancement mechanisms. (B) Schematics of a capacitance‐based microneedle sensor. (C) Optical image of the polydimethylsiloxane/carbonyl iron particles composed dielectric microneedles. Reproduced with permission.^78^ Copyright 2019, Royal Society of Chemistry.

In a capacitance‐based sensor, the microneedle part acts as the dielectric layer.^77^ The introduction of such a microneedle‐like structure can greatly improve the sensitivity of the capacitance‐based sensor. To theoretically explain this, the capacitance per unit area of a parallel‐plate capacitor is defined as $C\;=\;\frac{\varepsilon_{r}\varepsilon_{0}}{d}$ ($\varepsilon_{r}$: permittivity of the dielectric layer, $\varepsilon_{0}$: permittivity of the free space, d: distance between the parallel plates). When the dielectric layer is solid, the applied pressure only changes the plate distance and the relative permittivity remains constant; while with microneedles as the dielectric layer, the applied pressure can remove the air and deform the microneedles, leading to both the decreased distance and the varied permittivity (Figure 6A).^78^ Therefore, the capacitance variation is obviously improved. For experimental demonstration, Zhou et al. used polydimethylsiloxane containing carbonyl iron particles as the dielectric microneedles (Figure 6B,C), which behaved well in monitoring body movements such as elbow bending and relaxation, voice vibration, wrist pulse, finger bending and relaxation, standing, walking, and jumping.^78^ Besides, these capacitance‐based sensors also had demonstrated values in detecting neck pulse^79^ and breathing.^80^ In addition, to further improve the sensitivity and detection limit, Niu et al. designed a dielectric layer with two microneedle films facing each other and verified their high sensing performance.^81^

### 3.6 Others

**FIGURE 7:**
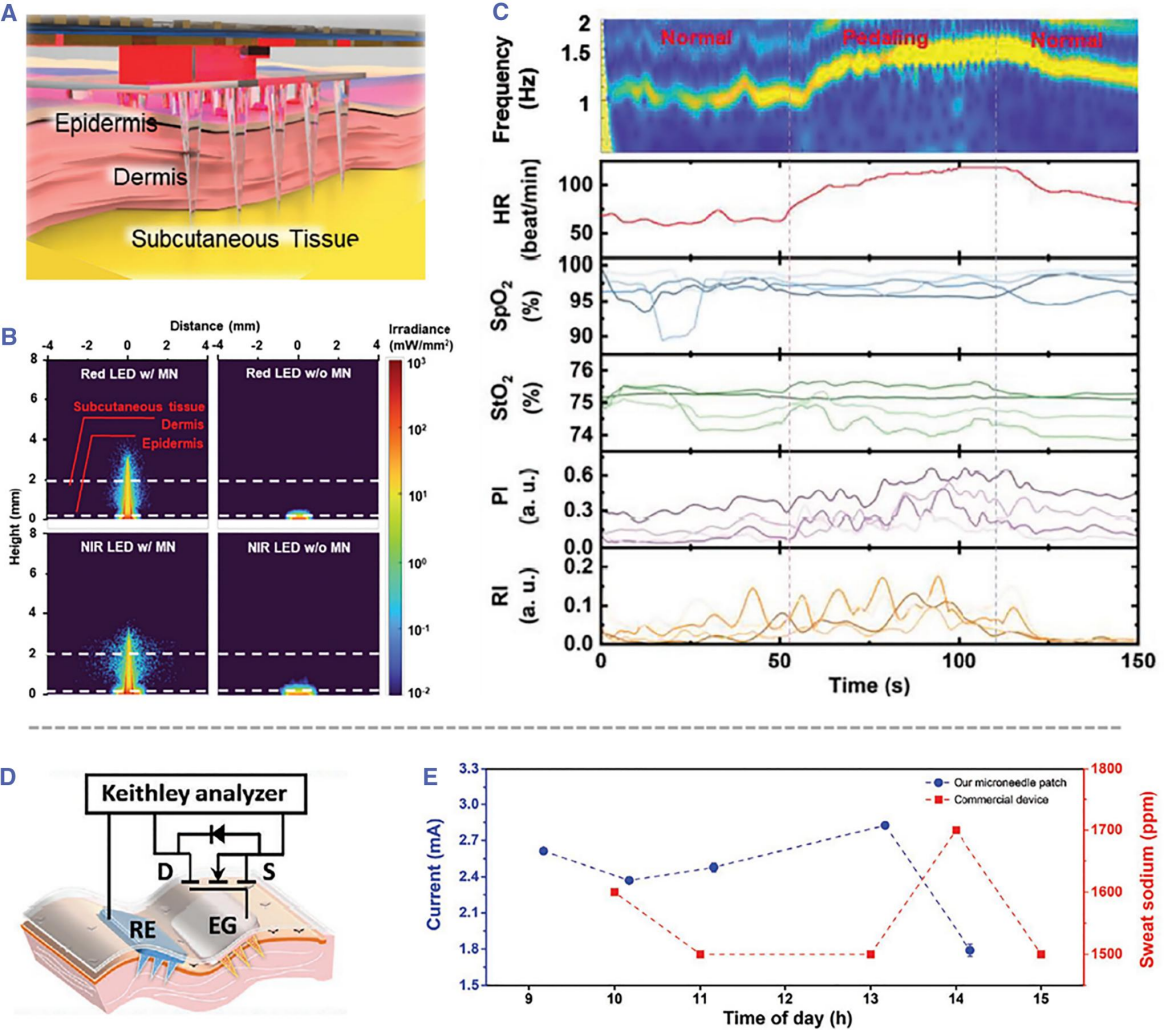
Other functional microneedles as sensing units. (A–C) Deep‐tissue sensing device dependent on microneedle waveguides: schematic of applying the device to the skin (A); simulation results of red and NIR light penetrating the skin tissue with and without microneedle waveguides, respectively (B); measurement results of multiple physiological signals (HR: heart rate, SpO_2_: pulse oximetry, StO_2_: tissue oximetry, PI: pulse magnitude and RI: respiratory magnitude) when subjects are cycling (C). Reproduced with permission.^82^ Copyright 2022, John Wiley and Sons. (D, E) Microneedle‐based extended gate field‐effect transistor biosensors: schematic of the composition of the biosensor (D); recording of interstitial fluid sodium concentrations by the biosensor in comparison with sweat sodium levels measured by commercial devices (E). Reproduced with permission.^83^ Copyright 2022, John Wiley and Sons.

Transparent microneedles can transmit light into deep tissue with minimal light attenuation (Figure 7A).^82^ Using poly(lactic‐co‐glycolic acid) and polyvinyl alcohol as the microneedle materials, it was found that the penetration depths of red light (639 nm) and NIR light (950 nm) were enhanced with the assistance of these microneedle waveguides by 10.16 and 6.89 times, respectively (Figure 7B). By incorporating the microneedle waveguides with light‐emitting diodes and photodiodes, the integrated sensor could efficiently measure multiple physiological signals, including pulse oximetry, tissue oximetry, pulse magnitude, and respiratory magnitude (Figure 7C).^82^ In addition, since microneedles can easily access analytes in interstitial fluids without extraction or any excessive methods, it would be ideal to combine them with a field‐effect transistor (FET) to build a household wearable biosensor. Such microneedle‐based extended gate FET biosensors contained a FET transducer and a sensing part that was composed of a microneedle extended gate, a microneedle reference electrode, and a pair of silver nanowire electrodes (Figure 7D).^83^ These biosensors were demonstrated to provide real‐time information of sodium concentrations in interstitial fluids and to report the results to smartphones/computers. Sodium fluctuation in interstitial fluids detected by the biosensors had a similar tendency, but a little in advance, to that in sweat detected by commercial devices, indicating the validation of the microneedle‐based FET biosensors (Figure 7E).^83^

In conclusion, polymer or conductive microneedles can collect molecules in interstitial fluids by swelling, reverse iontophoresis, or the specific recognition between targets and probes. With further integration of electrochemical sensors, impedance sensors, capacitance sensors, conductive electrodes, waveguide elements, field effect transistors, and other techniques, precise and sensitive detection can be successfully realized.

## 4 MECHANISM OF MICRONEEDLE‐BASED POWER UNITS

The shape geometry and flexibility of microneedle‐like structures can improve the performances of electric generators such as magnetoelectric^84^, ^85^ and triboelectric nanogenerators.^86^, ^87^ Because of this advantage, microneedles can act as power units of wearable electronics to sensitively perceive weak tactile signals and enable self‐powering. In this way, the service life of such microneedle‐powering sensors can be extended, and their user‐friendliness can be enhanced.

### 4.1 Magnetoelectric sensor

**FIGURE 8:**
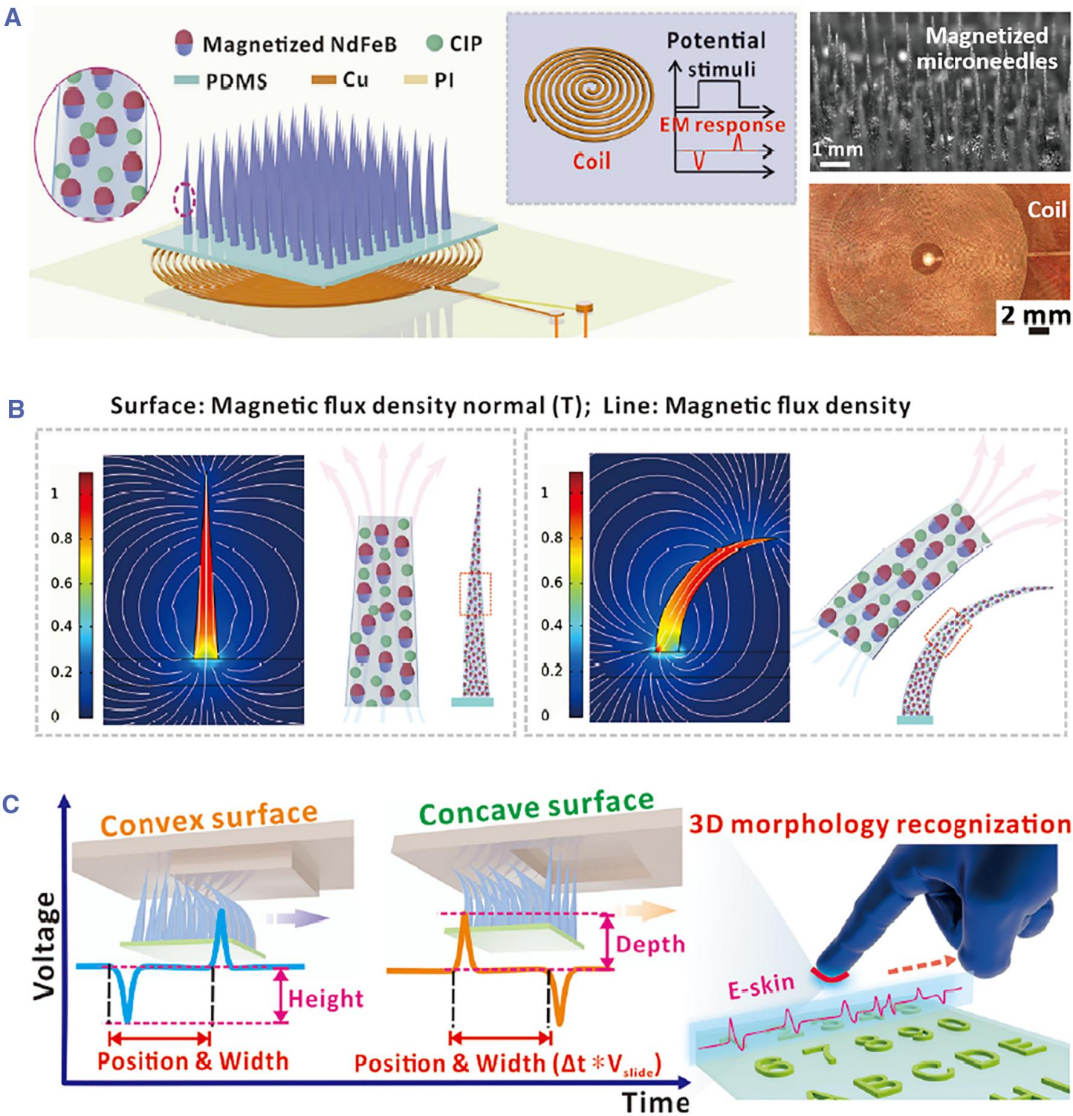
Microneedle‐integrated self‐powered magnetoelectric skins. (A) Schematics of the composition of the magnetoelectric skins that contain magnetized microneedles and underneath flexible coils, as well as optical images of the microneedles and coils. (B) Simulation results of magnetic field variation during the bending process of microneedles. (C) Principle of 3D morphology recognition via the magnetoelectric skins. Reproduced with permission.^85^ Copyright 2022, John Wiley and Sons.

Electromagnetic induction is the basic mechanism for magnetoelectric sensors.^84^ To be specific, an external force will lead to the relative movements of the electrical/magnetic parts, which in turn, cause the change of the magnetic flux. According to Faraday's law of electromagnetic induction, electrodynamic potential will be yielded by the altering magnetic flux, allowing for mechanoelectrical conversion and corresponding electrical outputs. Based on this principle, Zhou et al. integrated magnetized microneedles and underneath flexible coils for producing self‐powered magnetoelectric skins, as shown in Figure 8A.^85^ The simulation results in Figure 8B vividly displayed the variation of magnetic flux density during the force‐induced bending process of the microneedles. Thus, when the magnetoelectric skins moved along a convex or concave surface, the microneedles would bend or straighten, producing surface morphology‐determined voltage changes and thus realizing 3D topology recognition and tactile sensing (Figure 8C).^85^

### 4.2 Triboelectric sensor

**FIGURE 9:**
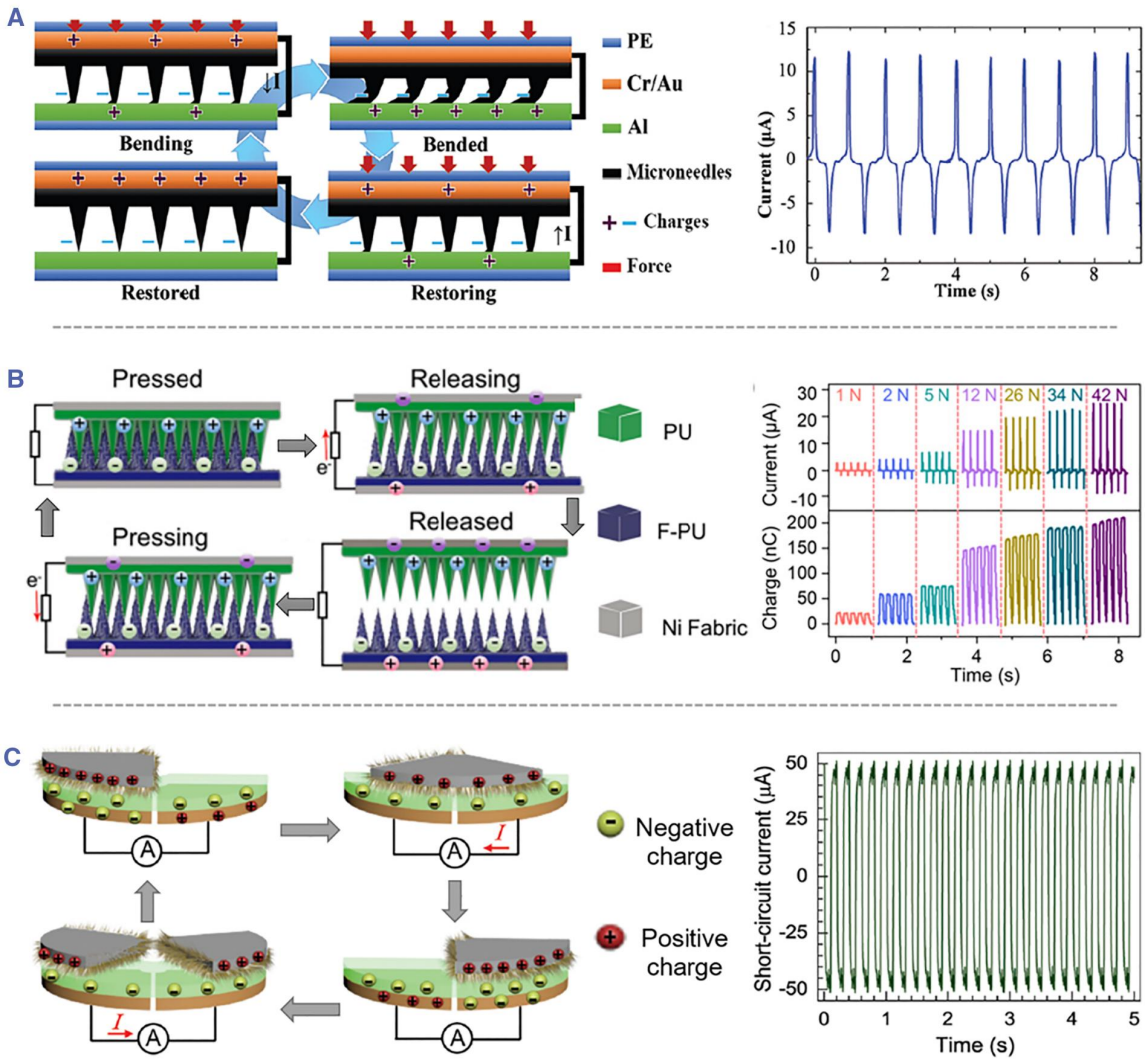
Charging cycles and performances of microneedle‐integrated triboelectric nanogenerators. (A) Vertical contact‐separation mode with only one microneedle friction layer and its short‐circuit current. Reproduced with permission.^90^ Copyright 2019, John Wiley and Sons. (B) Vertical contact‐separation mode with two microneedle friction layers and its short‐circuit current/transferred charge under different forces. Reproduced with permission.^91^ Copyright 2022, Elsevier. (C) Freestanding triboelectric‐layer mode and its short‐circuit current. Reproduced with permission.^92^ Copyright 2022, John Wiley and Sons.

The key reason for microneedle‐induced triboelectric performance enhancement lies in the high density and aspect ratio of microneedles, which increase the contact‐surface area as well as the friction behavior between the two triboelectric plates.^88^, ^89^ One typical triboelectric charging cycle is presented in Figure 9A and this process is accompanied by the bending and restoring of the microneedles.^90^ The microneedles are initially negatively charged, and both electrodes are positively charged. In the beginning, the applied external force causes the microneedles to rub against the bottom counterelectrode. Their contact‐surface area gradually increases along with the bending of microneedles. To maintain electrical neutrality and to re‐establish electrostatic balance, electrons are driven from the bottom counterelectrode to the top electrode, leading to a positive current. When the pressure is withdrawn, the bent microneedles restore, the contact‐surface area decreases, and the induced potential difference drives electrons toward the bottom counterelectrode, resulting in a negative current. In this way, alternating current flows can be generated by the microneedle‐based triboelectric sensor.^90^ To further improve the triboelectric performance, both the top and bottom electrodes can be integrated with microneedles. The reciprocating pressing and releasing behaviors between the two microneedle layers bring about the varying potential difference, which in turn induces electron flow between the two electrodes and produces alternating current flows (Figure 9B).^91^

In addition to the aforementioned vertical contact‐separation mode, microneedles can play a role in establishing triboelectric sensors in the freestanding triboelectric‐layer mode. For instance, Zhang et al. designed a disc‐shaped triboelectric nanogenerator that had a rotatable hairy friction layer and a pair of fixed fluorinated ethylene propylene (FEP) friction layers.^92^ Each of the FEP layers was connected with an electrode disc. At the outset, the hairy layer only contacted one of the FEP layers, and they were positively and negatively charged, respectively (Figure 9C). With the hairy layer rotating on the surfaces of the FEP layers, the potential difference occurred, and free electrons on the electrode discs were redistributed to maintain the electrostatic balance. As a result, periodically alternating currents were generated.^92^

Due to their sensitive mechanical‐electronic conversion ability, microneedle‐based triboelectric sensors can be applied for tactile perception and even intelligent human‐machine interactions.^93^ Besides, the triboelectric sensor fabricated by Wen and his coworkers was found to have a wide detection range (8 Pa to 71.85 kPa), high sensitivity in both low‐pressure and high‐pressure regions (6.66 kPa^−1^ and 0.79 kPa^−1^, respectively), fast response time (30 ms), and quick recovery (10 ms).^94^ Benefitting from these properties, the triboelectric sensor behaved well in monitoring human activities including swallowing and finger bending.^94^ In addition, Luo et al. designed a flexible triboelectric sensor.^95^ The researchers used microneedle‐shaped tea‐powder‐encapsulated polydimethylsiloxane as the negative friction layer and NaCl‐containing polyethylene oxide as the positive friction layer.^95^ This triboelectric sensor could rapidly and accurately record body movements, recognize speaking and yawning, and judge breathing intensity. Based on these competences, the triboelectric sensors further found practical applications in safety guarantee for drivers, where they could be attached to the safety belt or the neck of the driver to real‐time monitor driver status and degree of fatigue.^95^

In summary, microneedle structures can serve as a part of power units to improve the performances of magnetoelectric and triboelectric nanogenerators. This enables the self‐powering of wearable electronics and the perception of weak pressure and tactile signals. In this way, the microneedle‐based nanogenerator sensors can benefit motion sensing, electronic skins, human‐machine interaction, and so on.

## 5 APPLICATIONS OF MICRONEEDLE‐BASED SENSORS

Up to now, the microneedle‐integrated wearable electronics have been applied to human health management and disease prevention, with a large number of proof‐of‐concept progresses being achieved (Table 1). The microneedle‐based sensors are playing an increasingly important role in monitoring biophysical signals, including bioelectricity (e.g. brain waves), body movements (e.g. joint motions), breathing, blood oxygen, and so on.^5^, ^86^, ^87^, ^96^ On the other hand, these sensors perform well in detecting biochemical analytes in tissue interstitial fluids, such as physiological and pathological indicators (e.g. glucose and lactase) and disease biomarkers (e.g. cfDNA).^23^, ^51^, ^52^, ^97^, ^98^, ^99^

**TABLE 1:** Examples of applications of microneedle‐based sensors

	Signals/analytes	Microneedle materials	Detection mechanisms	Refs
Biophysical signals	Electroencephalogram	Polyimide polymer coated by Cr and Au film	Conductance‐based microneedle electrode	76
	Electromyogram	Medical‐grade stainless steel	Conductance‐based microneedle electrode	74
	Body movement (e.g. facial expression, speaking, swallowing, joint movement, gait)	Polydimethylsiloxane (PDMS) polymer containing carbonyl iron particles, carbon nanotube and carbon black; PDMS polymer containing galinstan	Resistance‐based microneedle sensor; capacitance‐based microneedle sensor; triboelectric microneedle sensor	71, 78, 94
	Breathing	PDMS polymer containing silver‐coated nickel magnetic particles; Ecoflex silicone containing Nd_2_Fe_14_B powders; PDMS polymer containing tea powders	Capacitance‐based microneedle sensor; magnetoelectric microneedle sensor; triboelectric microneedle sensor	80, 84, 95
	Voice	PDMS polymer coated with MXene (Ti_3_C_2_T_x_)	Resistance‐based microneedle sensor	69
	Pulse	PDMS polymer coated with poly(3,4‐ethylenedioxythiophene–poly(styrenesulfonate); polyvinyl alcohol/H_3_PO_4_ ionic gel	Resistance‐based microneedle sensor; capacitance‐based microneedle sensor	67, 77
	Oximetry	poly(lactic‐co‐glycolic acid) and polyvinyl alcohol polymer	Microneedle waveguide	82
Biochemical analytes	pH	Au deposited by polyaniline	Electrochemical microneedle sensor	59
	Small molecule (e.g. sodium, H_2_O_2_, lactate, alcohol, glucose)	Stainless steel coated by Au; poly glycidyl methacrylate; poly(methyl methacrylate) polymer immobilized with enzymes; silicon; polystyrene polymer	Reverse iontophoresis‐based interstitial fluid absorption; electrochemical microneedle sensor; extended gate field‐effect transistor microneedle sensor	52, 60, 65, 83
	Nucleic acid biomarker	Polymethyl vinyl ether‐alt‐maleic acid (PMVE/MA) polymer;	Reverse iontophoresis‐based interstitial fluid absorption; electrochemical microneedle sensor	51, 63
		CRISPR‐Cas9 activated Au‐carboxyl graphene‐coated PMVE/MA polymer		
	Peptide/protein biomarker	Silk fibroin; hollow acrylate polymer with catechol‐coated carbon paste as the core	Interstitial fluid absorption; electrochemical microneedle sensor	50, 64
	Drug concentration	Au decorated with aptamers	Electrochemical microneedle sensor	66

### 5.1 Biophysical signal monitoring

**FIGURE 10:**
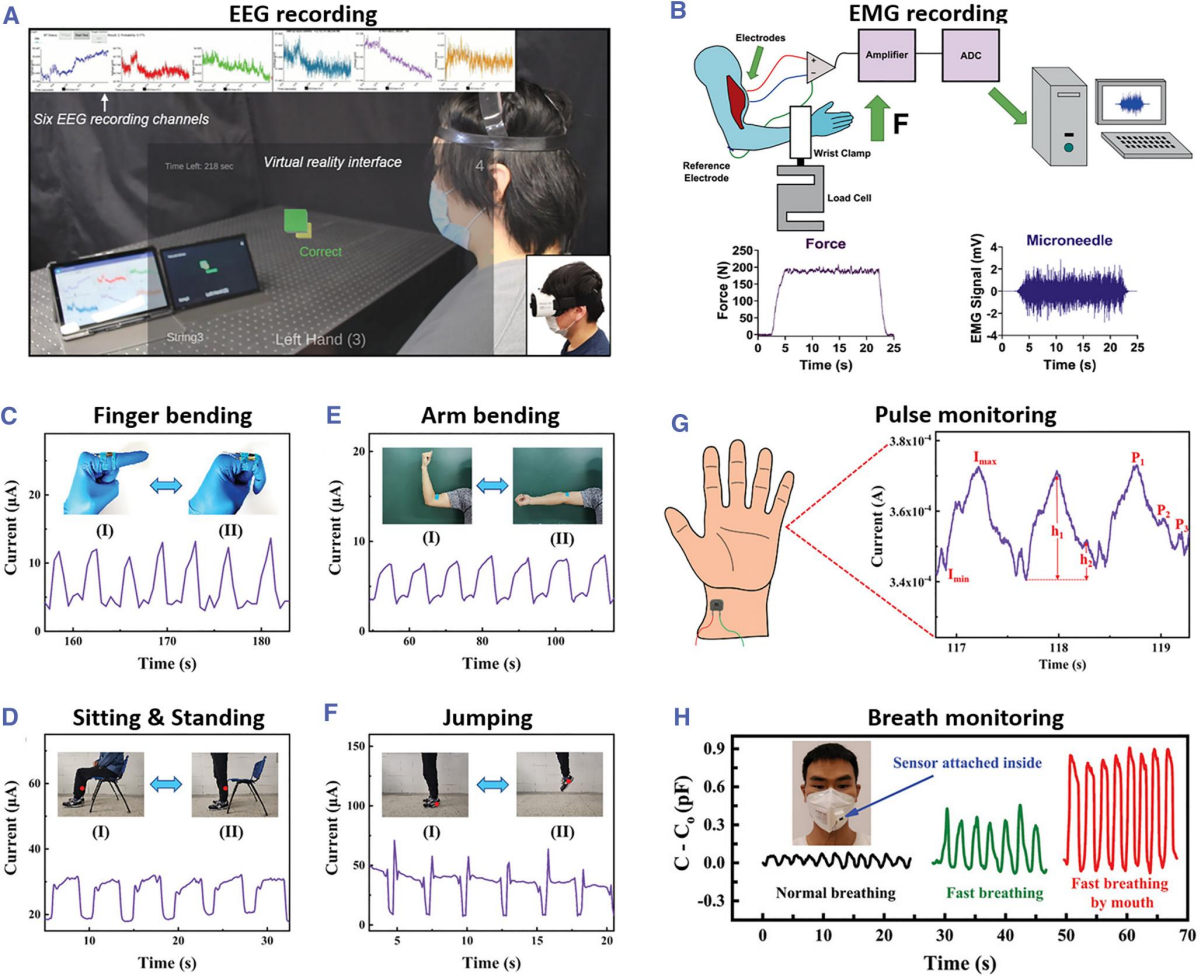
Microneedle‐integrated wearable electronics for monitoring biophysical signals. (A) Study setup of electroencephalogram (EEG) recording and VR game playing via the microneedle‐integrated scalp electronic system. Reproduced under terms of the CC‐BY license.^76^ Copyright 2021, The Authors, published by John Wiley and Sons. (B) Study setup of electromyogram (EMG) recording via the microneedle electrodes. Below are the force of a participant and the corresponding EMG recording. Reproduced with permission.^74^ Copyright 2020, John Wiley and Sons. (C–F) Current changes of the composite conductive microneedle sensor during finger bending (C), sitting and standing (D), arm bending (E), and jumping (F). Reproduced with permission.^72^ Copyright 2021, John Wiley and Sons. (G) Pulse monitoring via the composite conductive microneedle sensor and a typical wrist pulse wave. Reproduced with permission.^72^ Copyright 2021, John Wiley and Sons. (H) Capacitance changes of the capacitive sensor‐attached mask during different types of breathing. Reproduced with permission.^80^ Copyright 2020, John Wiley and Sons.

Conductance‐based sensors with microneedle electrodes can be used to gather bioelectrical signals. These sensors can be applied on the head or the muscles to record electroencephalogram or electromyogram. For example, Yeo and his coworkers developed a microneedle‐integrated scalp electronic system that had an ideal accuracy (93.22 ± 1.33% for 4 s of data) and a competitive information transfer rate (23.02 ± 1.11 bit min^−1^) (Figure 10A).^76^ This system also did well in brain‐machine interfaces. By wearing this, participants could play a rhythm‐type virtual reality (VR) video game and obtain high scores. Additionally, Krieger et al. used microneedle electrodes to measure human muscle activity (Figure 10B).^74^ Under the situation of 50% maximum voluntary contraction force, these microneedle electrodes showed a high signal‐to‐noise ratio (38.14 ± 5.35 dB) and a high median frequency (71.68 Hz). Remarkably, the signal‐to‐noise ratio (38 ± 5.02 dB) and high median frequency (68.78 Hz) of the microneedle electrodes were almost unchanged after they were applied to participants for up to 6 h, indicating their potential for long‐term electromyogram monitoring.^74^

For body movement recognition, a large variety of microneedle‐integrated wearable sensors, including resistance, capacitive, magnetoelectric, and triboelectric ones, have been developed. With the aid of these advanced sensors, people can get reliable and real‐time information about joint motions such as bending of knuckles, elbows, and knees, gaits such as jumping, walking, and standing, as well as facial expressions such as speaking, smiling, and laughing. For example, using the resistance‐based sensor composed of polydimethylsiloxane/carbon black/multiwalled carbon nanotubes/nano‐copper composite conductive microneedles, Li et al. achieved sensitive pressure detection and accurate movement identification (Figure 10C–F).^72^ To be specific, the detection range, sensitivity, response time, and detection limit of this sensor were 0‐396 kPa, 212 kPa^−1^, 32 ms and 7.69 Pa, respectively. Besides, after 5000 compression cycles, such sensors still showed high stability and consistency.^72^

Furthermore, microneedle‐integrated wearable sensors can contribute a lot to detecting pulse waves, breathing, blood oxygen, and many other biophysical signals. For instance, a resistance‐based sensor had the ability to record pulse waves in real time and obtain pulse information, such as vibration intensity, characteristic peaks, and periods (Figure 10G).^72^ It was found that the wrist pulse vibration intensity was enhanced (from 10, 26.1 to 26.7 μA) when the applied pressures increased (from 9, 15, to 21 kPa). The three characteristic peaks, which were the main pulse crest, the front wave of the heavy pulse, and the heavy pulse, could be seen from the recorded pulse waves, and the number of pulse cycles per minute could be counted.^72^ Besides, a capacitance‐based sensor was attached to a mask to monitor the pressure signals resulting from normal breathing, fast breathing by the nose, and fast breathing by the mouth (Figure 10H).^80^ Based on the amplitude and frequency of capacitance variations on the sensor, the three respiratory patterns could be easily distinguished. In addition, heart rate, pulse oximetry, and tissue oximetry could be measured by a microneedle waveguide‐based biosensor.^82^ When the subject was riding a bike, the recorded heart rate was significantly increased, with the pulse oximetry and tissue oximetry remained at ∼98% and ∼75%, respectively, indicating that pedaling was aerobic training.^82^

### 5.2 Biochemical analyte detecting

**FIGURE 11:**
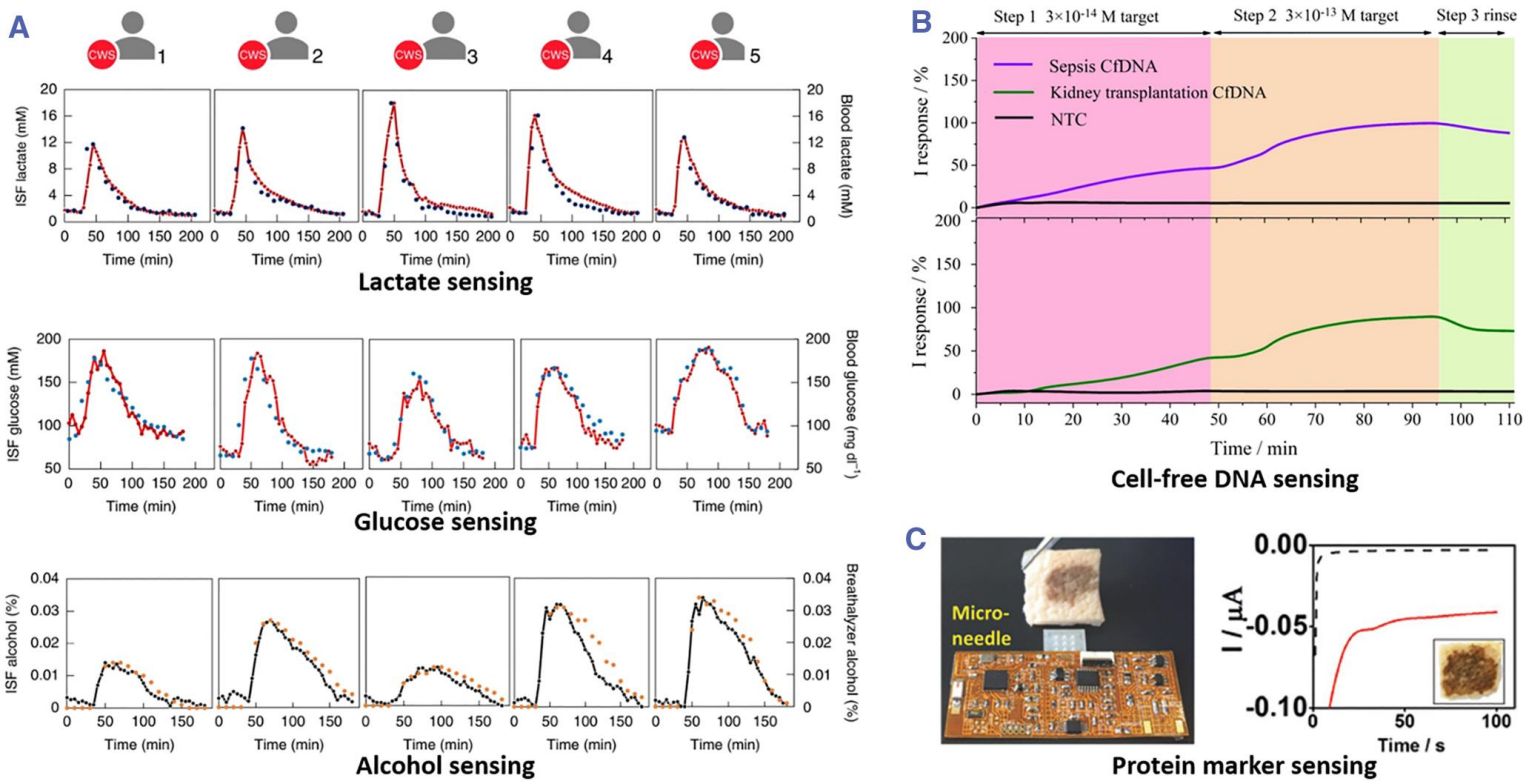
Microneedle‐integrated wearable electronics for detecting biochemical analytes. (A) Above: Lactate detection results from the fully‐integrated multiplex wearable microneedle system (dotted lines) in comparison with blood measurements (dots) of five subjects doing intermittent high‐intensity exercise. Middle: glucose detection results from the microneedle system (dotted lines) in comparison with blood measurements (dots) of five subjects consuming food. Below: alcohol detection results from the microneedle system (dotted lines) in comparison with breathalyzer measurements (dots) of five subjects drinking wine. Reproduced with permission.^65^ Copyright 2022, The Authors, published by Springer Nature. (B) Recording of sepsis‐related cfDNA and kidney transplantation‐related cfDNA via the wearable CRISPR‐Cas9 microneedle system. Reproduced under terms of the CC‐BY license.^63^ Copyright 2022, The Authors, published by Springer Nature. (C) Tyrosinase monitoring of an in vitro porcine skin model via the microneedle‐integrated wireless sensor. The Black dot line and the red line indicate the amperometric response of the sensor before and after interacting with the model, respectively. Reproduced with permission.^64^ Copyright 2018, John Wiley and Sons.

Regular physical examination is helpful to maintain physical health. Microneedle‐integrated wearable electronics can accurately and conveniently detect physiological or pathological indicators in interstitial fluids and clearly display detection results, making household body check‐up possible. To list a few, pH, sodium concentrations, hydrogen peroxide, glucose, lactase, and alcohol, etc. have been precisely detected by microneedle reverse iontophoresis sensors,^52^ microneedle electrochemical sensors,^22^ or microneedle FET sensors.^83^ Recently, Tehrani et al. developed a fully‐integrated multiplex wearable microneedle system and evaluated its ability to continuously sense metabolites in interstitial fluids in real life.^65^ Three common metabolites, glucose, lactase, and alcohol, were chosen for the experiments (Figure 11A). The fully sterilized microneedle sensors were fixed on participants via double‐sided medical tape, with an extra piece of tape covering their surfaces. They could be worn for long periods of time or only when participants wanted to check their state of health, depending on actual needs and doctor's orders. Such a sensing system showed a wide detection range (0‐40 mM for glucose, 0‐28 mM for lactate, and 0‐100 mM for alcohol) and a low detection limit (0.32 mM for glucose, 0.15 mM for lactate, and 0.50 mM for alcohol). Notably, the detection results from this sensing system exhibited high consistency and a short lag time with reference measurements by traditional methods,^65^ indicating its reliability and practicability.

Microneedle‐integrated wearable electronics can facilitate early diagnosis, due to their capability to capture and sense disease‐related biomarkers, such as nucleic acids and protein markers. In this regard, microneedle‐based electrochemical biosensors play an important role. For nucleic acid biomarker detection, Yang et al. presented a wearable CRISPR‐Cas9 microneedle system for real‐time detection of cfDNA from Epstein‐Barr virus (EBV), sepsis, and kidney transplantation (Figure 11B).^63^ Taking the EBV cfDNA as an example, the entire monitoring time was less than 75 min, the detection limit was 1.2 fM, the sensitivity under interferences was 3 × 10^−14^ M, and the detection stability and reproducibility could remain for 12 days. Besides, for protein biomarker detection, Wang and his coworkers used microneedle‐integrated wireless sensors to monitor tyrosinase, a tumor biomarker, for melanoma screening (Figure 11C).^64^ The output signals of such sensors were found to have good linearity to tyrosinase concentrations with *R* ^2^ > 0.990. These sensors also showed good reproducibility with Relative Standard Deviation (RSD) < 10% (*n* = 3) and ideal stability with RSD = 1.1% after 7 days at room temperature.^64^

To sum up, microneedle‐integrated wearable electronics behave satisfactorily in proof‐of‐concept experiments. They have been demonstrated to make a difference in monitoring biophysical signals, including electroencephalogram, electromyogram, body movement, breathing, voice, pulse and oximetry, as well as detecting biochemical analytes, such as pH, small molecules, nucleic acids, peptides/proteins, and drug concentrations. The next step can be applying these microneedle‐based sensors to practical scenarios and evaluating their sensitivity, accuracy, and reliability in real life.

## 6 CONCLUSION AND OUTLOOK

This review briefly summarizes the mechanisms, designs, advantages, and applications of microneedle‐based wearable electronics. Microneedle structures can act as the sensing part for wearable electronics. Benefitting from their abilities of interstitial fluid absorption, biomarker accumulation, and easy decoration, microneedles can help the biosensors to sensitively and accurately detect metabolites and biomarkers. Also, the versatility, compatibility, and high specific surface area of microneedle structures can greatly improve the sensing performances of electrical biosensors. Additionally, microneedle structures contribute to the fabrication of self‐powered wearable electronics. Due to their shape geometry and flexibility, the sensitivity and electric generation efficiency of microneedle‐based sensors are dramatically enhanced. Up to now, a variety of microneedle‐assisted magnetoelectric or triboelectric sensors have been developed for strain and pressure perception. It is believed that these microneedle‐integrated wearable electronics can provide a promising road for intelligent household physical examination, individualized health care, disease prevention, and early diagnosis. Particularly, the application of microneedle‐integrated wearable electronics in recording body movements, pulses, and small molecular metabolites like glucose has gone through lots of proof‐of‐concept experiments and even human trials and is expected to replace traditional detection methods.

There is still a lot of space for the improvement of microneedle‐integrated electronics. Firstly, it would be fine to include novel soft polymers^100^, ^101^, ^102^, ^103^, ^104^ in the material compositions to endow the microneedle biosensors with breathable, stretchable, conformable, responsive, or other additional functions. Notably, degradable, recyclable, or even compostable materials are with high anticipation to make these sensors environmentally friendly. Secondly, the sensing unit of the microneedle biosensors can be updated by introducing newly emerged biorecognition and assay techniques^3^, ^105^, ^106^, ^107^, ^108^ such as molecular imprinting, CRISPR‐Cas toolbox, nanozymes, and so on. In addition, it will be a great progress to achieve multimodal or multiplexed detection by fusing different transducer types or mixing different probes into the same microneedle‐based sensing system. Moreover, to ensure the long‐term stability and reliability of the microneedle biosensors, more human tests are required, and the testing time should be prolonged, which will promote the clinical and market translation. Lastly, with the advent of the digital era, it is a trend to link the microneedle biosensors to information technologies,^109^, ^110^, ^111^, ^112^ including cloud or fog computing, machine learning, data mining, neural network, etc., to establish extremely large databases and realize personalized medicine.

## AUTHOR CONTRIBUTIONS

Yuanjin Zhao conceived the topic. Xiaoxuan Zhang organized the content, wrote the manuscript, and arranged the figures. Minhui Lu and Xinyue Cao checked the grammar and figures. Yuanjin Zhao revised the manuscript.

## CONFLICT OF INTEREST STATEMENT

The authors declare no conflict of interest. Yuanjin Zhao is a member of the *Smart Medicine* editorial board.
